# RNAi-based functional elucidation of *PtrPRP*, a gene encoding a hybrid proline rich protein, in cold tolerance of *Poncirus trifoliata*

**DOI:** 10.3389/fpls.2015.00808

**Published:** 2015-09-29

**Authors:** Ting Peng, Mao-Mao Jia, Ji-Hong Liu

**Affiliations:** ^1^Key Laboratory of Horticultural Plant Biology, College of Horticulture and Forestry Sciences, Huazhong Agricultural University, WuhanChina; ^2^National Navel Orange Engineering Research Center, College of Navel Orange, Gannan Normal University, GanzhouChina

**Keywords:** cold stress, *Poncirus trifoliata*, hybrid proline-rich protein, RNA interference, ROS

## Abstract

Hybrid proline-rich proteins (HyPRPs) have been suggested to play important roles in various plant development and stress response. In this study, we report the cloning and functional analysis of *PtrPRP*, a HyPRP-encoding gene of *Poncirus trifoliata*. PtrPRP contains 176 amino acids, among which 21% are proline residues, and has an 8-cysteine motif (8 CM) domain at the C terminal, a signal peptide and a proline-rich region at the N terminal. *PtrPRP* is constitutively expressed in root, stem and leaf, with the highest expression levels in leaf. It was progressively induced by cold, but transiently upregulated by salt and ABA. Transgenic *P. trifoliata* plants with knock-down *PtrPRP* by RNA interference (RNAi) were generated to investigate the role of *PtrPRP* in cold tolerance. When challenged by low temperature, the *PtrPRP*-RNAi plants displayed more sensitive performance compared with wild type (WT), as shown by higher electrolyte leakage and malondialdehyde content. In addition, the RNAi lines accumulated more reactive oxygen species (ROS) and lower levels of proline relative to WT. These results suggested that PtrPRP might be positively involved in cold tolerance by maintaining membrane integrity and ROS homeostasis.

## Introduction

Plants are frequently challenged with a variety of abiotic stresses, among which cold, at either freezing, or chilling regimes, constitutes an important factor leading to adverse impacts on plant growth, development and yield potential. Chilling and freezing stresses lead to physiological or structural alterations, such as elevation of reactive oxygen species (ROS), imbalance of osmotic pressure, and formation of ice crystals. All of these unfavorable situations will result in deterioration of membrane integrity, impairment of cell viability and eventually lead to cell death when they are not precisely coped with (Xiong and Zhu, 2002; Einset et al., 2007; Peng et al., 2012).

It has been well known that to survive under cold plants experience a cascade of physiological and biochemical changes, such as accumulation of various proteins or soluble compounds, and alteration of a series of metabolic reactions (Wilson and Cooper, 1994; Knight et al., 1996; Fowler et al., 1999; Welling and Palva, 2006; Huang et al., 2011). In addition, accumulating evidences show that extensive reprogramming of a cohort of cold-responsive genes is an elegant strategy for the plants to adapt to the harsh environments (Stockinger et al., 1997; Zarka et al., 2003; Nakashima and Yamaguchi-Shinozaki, 2006; Shi et al., 2015). These genes are generally classified into two major categories, functional proteins, and regulatory proteins, which play either direct protective or regulatory roles in stress tolerance (Yamaguchi-Shinozaki and Shinozaki, 2005). For example, the functional proteins participate in stabilization of membrane integrity, maintenance of enzyme activity, physical structures of cellular components, which are critical factors contributing to enhance stress tolerance in plants (Walker et al., 2010). Genetic manipulation of the cold-responsive genes has been suggested to serve as an alternative approach for generating transgenic plants with enhanced stress tolerance (Huang et al., 2013). However, it is worth mentioning that functional characterization of a stress-responsive gene is required before it can be efficiently manipulated.

Hybrid proline-rich proteins (HyPRPs) are a subset of proline-rich proteins (PRPs) specific to seed plants; however, they do not contain the domain typical of PRPs (Josè-Estanyol et al., 2004; Priyanka et al., 2010; Xu et al., 2011). Based on the position and number of cysteine residues at C terminal, HrPRPs can be categorized into two major groups, in which Class A has 4–6 cysteine residues, while Class B contains a conserved eight-cysteine-motif (8 CM) at C terminal, a repetitive proline-rich N-terminal domain (PRD) and a signal peptide in front of the PRD (Josè-Estanyol and Puigdomènech, 2000; Josè-Estanyol et al., 2004; Battaglia et al., 2007; Dvořáková et al., 2007; Neto et al., 2013). HyPRPs have been suggested to play important biological roles in various processes, including plant cell elongation, ontogeny, and morphogenesis of different organs, defenses against viral or fungal pathogens (He et al., 2002; Holk et al., 2002; Dvořáková et al., 2012). In addition, HyPRPs of *Arabidopsis thaliana*, *Brassica napus*, *Medicago sativa*, *Glycine max*, and *M. truncatula* have been also suggested to be involved in responses to biotic and abiotic stresses (Deutch and Winicov, 1995; He et al., 2002; Bouton et al., 2005; Zhang and Schläppi, 2007). Nevertheless, so far little knowledge is available on the role of HyPRPs in stress tolerance of perennial plants, such as *Poncirus trifoliata*.

*Poncirus trifoliata* (L.) Raf. is extremely cold hardy when it is fully cold acclimated. In a previous work, suppression subtractive hybridization was employed to unravel cold-responsive genes of valuable significance for engineering cold tolerance (Peng et al., 2012). One of the ESTs draws our attention as its expression level was elevated over 70-folds under cold; the EST was later annotated to encode a HyPRP. However, whether it plays a role in cold tolerance remains undetermined. As a follow-up and continuum of our earlier work, in this study we report the isolation and functional analysis of this gene, designated as *PtrPRP*, in cold tolerance by RNA interference (RNAi). Transcript levels of *PtrPRP* were enhanced by abiotic stresses, but the response to cold was extremely dramatic. Knock-down of *PtrPRP* in trifoliate orange by RNAi led to enhanced cold sensitivity. We further demonstrated that the RNAi lines accumulated more ROS and malondialdehyde (MDA), but lower levels of proline. Our data indicate that *PtrPRP* is a cold-responsive gene that plays an essential role in cold tolerance.

## Materials and Methods

### Plant Materials and Stress Treatments

Three-month-old trifoliate orange seedlings were grown in a growth chamber (25°C) with a photoperiod regime of 8 h dark/16 h light (light intensity is about 100 μmol m^-2^ s), and an relative humidity of 60–70%. The seedlings were well watered before they were subjected to various stresses. For cold treatment the seedlings were placed in the chamber set at 4°C for 6 d, followed by transfer to 25°C for recovery. The leaves were sampled at 0, 6, 24, 72, and 144 h after cold treatment and 6 h after recovery. Dehydration was imposed by placing the seedlings on a clean bench under ambient environment for 6 h and then shifted to water for 0.5 h. The leaves were collected at 0, 0.5, 1, 3, and 6 h after dehydration and 6 h after rehydration. For salt stress, the seedlings were treated with 200 mM NaCl solution for up to 144 h and then shifted to water for another 6 h; the leaves were sampled at 0, 6, 24, 72, and 144 h of salt treatment and after 6 h of recovery in water. In addition, the seedlings were treated with 100 μM ABA for 0, 6, 12, 24 h, and 48 h, followed by transfer to water for another 6 h; the leaves were sampled at the designated time points. The samples were immediately frozen in liquid nitrogen and stored at -80°C until further use.

### Gene Isolation and Sequence Analysis

Rapid amplification of cDNA ends (RACEs) was employed to obtain full-length cDNA of *PtrPRP*. For this purpose, gene-specific primers for 5′-RACE and 3′-RACE (**Table 1**) were designed based on the EST sequence identified in the previous work (Peng et al., 2012). RACE-PCR was carried out using the SMART^TM^ RACE cDNA Kit (Clontech, USA). Total RNA was extracted with RNAiso Plus (TaKaRa, Japan) according to the manufacturer’s instructions. First strand cDNA was synthesized via PrimeScript^®^ RT Reagent Kit With gDNA Eraser (TaKaRa) following the user’s manual. The 5′-RACE and 3′-RACE PCR products were sequenced and analyzed, and then merged with the original EST to get a single sequence, which was verified using RT-PCR with a pair of full-length primers (PtrPRP-S/PtrPRP-A, **Table 1**). Molecular weight (MW) and isoelectric point (pI) of the protein were predicted on ExPASy^1^. Phylogenetic analysis was constructed by Phylogeny.fr online software ^2^ (Dereeper et al., 2008) and MEGA4.0. Multiple alignments of the HyPRPs were performed by ClustalX program with defaulted settings and displayed by Jalview^3^. Signal peptide was predicted with SignalP4.1 Server^4^ and iPSORT^5^ (Bannai et al., 2002).

**Table 1:** Oligonucleotide primers used in this study.

Name	Primer sequences (5′ – 3′)
5′-RACE	CGCCAAACCGAAATGTGCTCTGATA
3′-RACE	TAGTGTGAGATACCCACCGCC
PtrPRP-S	ATGGGAAAATATCAATTAGC
PtrPRP-A	TTAAGCAGGACACTGAAATCC
PtrActin-S	CATCCCTCAGCACCTTCC
PtrActin-A	CCAACCTTAGCACTTCTCC
PtrPRP-q-S	ACCGATTGTAAAGACGCCAC
PtrPRP-q-A	CACCGAGTTTGAGAGCATCA
PtrPRP-L-S	GAAGATCTATGGGAAAATATCAATTAGC
PtrPRP-L-A	GACTAGTAGCAGGACACTGAAATCC
PtrPRP-attB-S	GGGACAAGTTTGTACAAAAAAGCAGGCTATGGGAAAATATCAATTAGC
PtrPRP-attB-A	GGGGACCACTTTGTACAAGAAAGCTGGGTCTTAGGGCTTGGTCCGTTA
NPTII-S	AGACAATCGGCTGCTCTGAT
NPT II-A	TCATTTCGAACCCCAGAGTC
PtrPRP-s-S	ATGGGAAAATATCAATTAGC
PtrPRP-s-A	TTAAGCAGGACACTGAAATCC

### Gene Expression Analysis by Quantitative Real-time RT-PCR (qPCR)

Total RNA extraction and cDNA synthesis were performed as mentioned above. Expression of *PtrPRP* under the stresses and in different tissues was assessed by qPCR, which was carried out with the SYBR^®^ Green PCR kit (TaKaRa) on a LightCycler 480 Real-Time System (Roche). PCR solution, in a total volume of 10 μl, contained 5 μl of 2 × SYBR Premix Ex Taq (Tli RNaseH Plus), 50 ng of cDNA, 0.25 μM of each primer (PtrPRP-q-S/A). The reaction cycles were 95°C for 30 s, and 40 cycles of 95°C for 5 s, 56°C for 10 s, and 72°C for 15 s. Each reaction was repeated at least three times, and ^ΔΔ^CT method was applied to calculate relative expression levels. The *Actin* gene of trifoliate orange was used as a reference control and analyzed in parallel with specific primers (**Table 1**) to normalize the expression levels.

### Subcellular Localization of PtrPRP

To determine subcellular localization of PtrPRP, the full-length *PtrPRP* cDNA without stop codon was amplified using primers (PtrPRP-L-S/A) containing restriction sites of *Bgl*II and *Spe*I. The PCR products were purified with AxyPrep^TM^ DNA Gel Extraction Kit (Axygen scientific, USA), digested with *Bgl*II and *Spe*I and subcloned into the pCAMBIA1302 vector containing a GFP reporter gene, under the control of *CaMV 35S* promoter. The resultant fusion construct PtrPRP::GFP and the control vector (GFP) were separately introduced into onion epidermis via *Agrobacterium*-mediated transformation as described by Peng et al. (2014), followed by visualization of green fluorescence under a confocal microscope (FV1000; Olympus,Tokyo, Japan) or a fluorescence microscope (Nikon 90i).

### Generation and Identification of RNAi Plants

To generate *PtrPRP*-RNAi plants, a 253-bp cDNA fragment of *PtrPRP* was amplified using a pair of primers (PtrPRP-attB-S/A) and introduced into pHELLSGATE2 through BP recombination reactions (Invitrogen, Japan). The RNAi vector was introduced into *A. tumefaciens* strain GV3101. *Agrobacterium*-mediated transformation of trifoliate orange was performed according to Fu et al. (2011). Kanamycin-resistant shoots were identified by genomic PCR using primers specific to neomycin phosphotransferase II (NPTII-S/A) and the 253-bp sequence (**Table 1**). Examination of *PtrPRP* expression was carried out by semi-quantitative RT-PCR according to Shi et al. (2010) except using specific primers (PtrPRP-s-S/A, **Table 1**). QRT-PCR was also used to confirm the expression of one transgenic line, as done mentioned above. *Actin* gene was used as the reference gene. The positive transgenic plants were vegetatively propagated to obtain enough plants that were used for the subsequent experiments.

### Cold Tolerance Assessment and Physiological Measurements

Uniform and healthy 3-month-old plants of wild type (WT) and two RNAi lines were used for cold treatment. Two days after sufficient watering, the plants were placed in a growth chamber set at 0°C and kept for 48 h without light in order to avoid light-induced oxidative stress under cold treatment. The leaves were sampled after completion of the chilling treatment and used for measurement of electrolyte leakage (EL), MDA, ROS, and proline.

Measurement of EL was conducted according to Peng et al. (2012). In brief, the sampled leaves were immersed in 20 mL of double distilled water (ddH_2_O), while the control tube contained only 20 mL of ddH_2_O. The tubes were gently shaken for 2 h on a shaker (QB-206, Qilinbeier, China) at room temperature; the EL of sample (C_1_) and control (CK_1_) were then measured on a DSS-307 conductivity meter (SPSIC, China). The tubes were then boiled for 10 min, and cooled down at room temperature before measurement of the EL (C_2_ and CK_2_). A relative conductance was calculated by C (%) = (C_1_ – CK_1_)/(C_2_ – CK_2_) × 100.

Proline content was determined according to Zhao et al. (2009) with minor modification. The leaf tissues (about 0.5 g) were extracted in 5 mL of 3% sulphosalicylic acid at 95°C for 10 min. After cooling down, the homogenate was filtered and 2 mL of supernatant was transferred to a new tube containing 2 mL of acetic acid and 2 mL of acidified ninhydrin reagent. After 30 min of incubation in boiling water, 4 mL of toluene was added to the tubes and vortexed for 30 s. The absorbance of the toluene layer was colorimetrically determined at 520 nm. Protein concentration was determined based on the method reported by Bradford (1976).

Accumulation of H_2_O_2_ and O_2_^-^, two major types of ROS, was assayed using histochemical staining with 3, 3′-diaminobenzidine (DAB) and nitrotetrazolium blue chloride (NBT), respectively. The leaves sampled after 48 h of chilling treatment were immediately immersed in 1 mg mL^-1^ freshly prepared NBT or DAB solution at ambient temperature, until blue or brown precipitates were observed. The stained leaves were then bleached in concentrated ethanol, and kept in 70% ethanol. Quantitative measurement of H_2_O_2_ was also carried out using a specific detection kit based on the manufacturer’s instructions (Nanjing Jiancheng Bioengineering Institute, China).

Malondialdehyde content, expressed as nmol/mg protein, was measured using a detection kit specifically designed for MDA quantification (Nanjing Jiancheng Bioengineering Institute, China) based on the manufacturer’s instructions. Protein concentration was determined based on the method reported by Bradford (1976).

### Statistical Analysis

The data were analyzed using analysis of variance (ANOVA), and statistical difference between WT and transgenic lines was compared, taking *P* < 0.05 as significant.

## Results

### Isolation of PtrPRP from *P. trifoliata*

In a previous work, an EST annotated as PRP was fished out by SSH-based screening of a cold-treated cDNA library of *P. trifoliata* (Peng et al., 2012). As the whole genome sequence information of *P. trifoliata* is unavailable at this time, we employed RACE to isolate the full-length cDNA of this gene. For this purpose, 5′-RACE and 3′-RACE PCR were carried out using primers designed base on the original EST, leading to amplification of two fragments of 554 and 802 bp, respectively, which were shown to be homologous to known PRP genes by Blastn against NCBI. Assembly of the two RACE sequences and the original EST resulted in generation of a full-length sequence of 849 bp in size, which was further verified by PCR to be correct in the sequence. The sequence displayed 74% of identity to PRP gene of *Solanum palustre*; so the gene was designated as *PtrPRP* (*Poncirus trifoliata Proline-Rich Protein*). The gene has been deposited in the NCBI database under the accession number of KF171887.1.

### Sequence Analysis of PtrPRP

**FIGURE 1:**
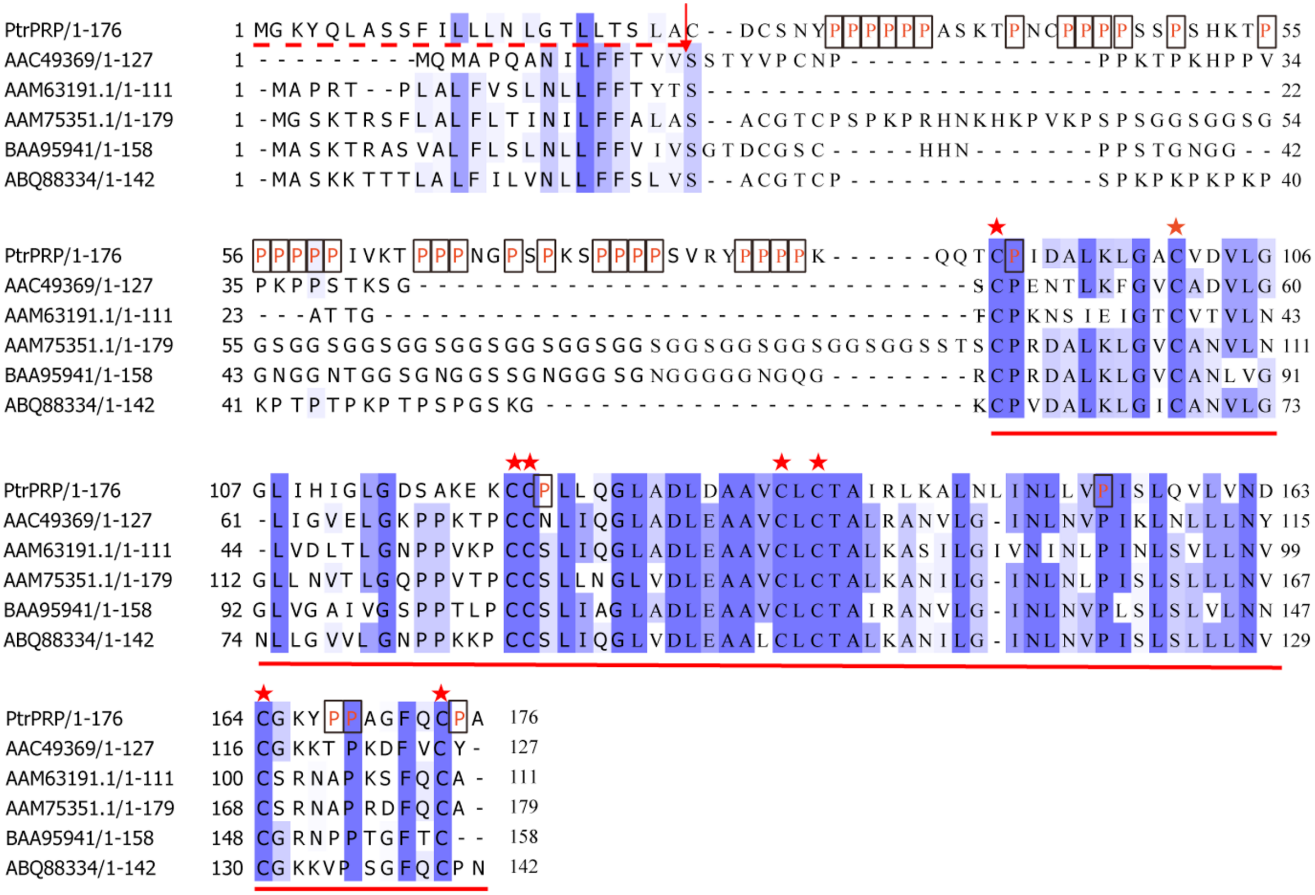
**Sequence alignment between PtrPRP and HyPRPs from other plants.** Prolines (P) residues in PtrPRP are marked in red letter and boxed. The signal peptide is indicated with dotted red line at the N-terminal, and the arrow indicates the cleavage site. Identical and highly conserved residues are shaded in dark and light blue, respectively. The conserved 8 CM domain at the C-terminal is underlined, while the cysteine residues are marked by asterisks. GenBank accession numbers for the HyPRPs are AGW00930.1 (PtrPRP, *Poncirus trifoliata*), AAC49369 (*Phaseolous vulgaris*), AAM63191.1 (*Arabidopsis thaliana*), AAM75351.1 (*Glycine max*), BAA95941 (*Nicotiana tabacum*), ABQ88334 (*C. annuum*).

**FIGURE 2:**
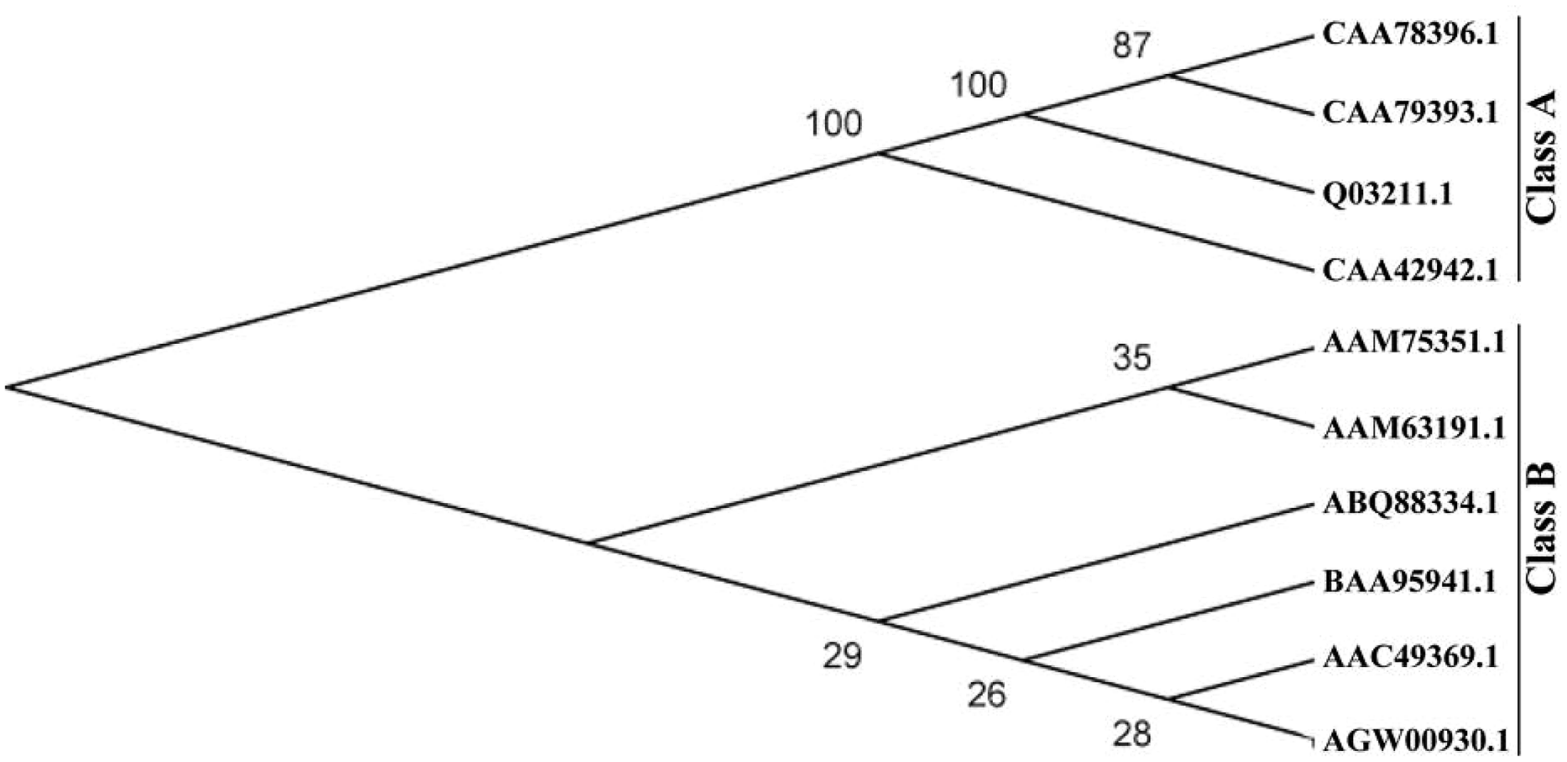
**A phylogenetic tree constructed using proline-rich proteins (PRPs) of trifoliate orange and other plants, including CAA78396.1 (*N. tabacum*), CAA78393.1 (*N. tabacum*), Q03211.1 (*N. tabacum*), CAA42942.1 (*Phaseolus vulgaris*), AGW00930.1 (PtrPRP, *Poncirus trifoliata*), AAC49369 (*Phaseolous vulgaris*), AAM63191.1 (*A. thaliana*), AAM75351.1 (*G. max*), BAA95941 (*N. tabacum*), and ABQ88334 (*C. annuum*).** The neighbor-joining tree was generated with MEGA4.0 from 1000 bootstrap replicates.

The full-length *PtrPRP* cDNA contained a 531-bp open reading frame (ORF) encoding 176 amino acids, 21% of which were proline residues (**Figure 1**). PtrPRP had a predicted molecular mass of 18.242 kD and an isoelectric point of 8.65. In addition, a signal peptide composed of 24 amino acids was observed at the N terminal. In order to reveal the relationship between PtrPRR and PRP proteins from other plants, we constructed a phylogenetic tree using deduced amino acid sequences of PtrPRP and PRPs from other plants. PRPs were evolutionarily divergent in plant. However, PtrPRP was not clustered into the five groups that have been established by earlier studies (data not shown). Therefore, we assume that PtrPRP might be a HyPRP. Alignment with PtrPRP and HyPRPs from *Phaseolous vulgaris*, *A. thaliana*, *G. max*, *Nicotiana tabacum*, and *Capsicum annuum* revealed that PtrPRP has the conserved 8 CM domain at the C terminal (**Figure 1**), which is an important signature for a HyPRP, indicating that PtrPRP was actually a typical HyPRP. Besides, the phylogenetic tree indicated that PtrPRP was a Class B HyPRP (**Figure 2**).

### Subcellular Localization of PtrPRP

**FIGURE 3:**
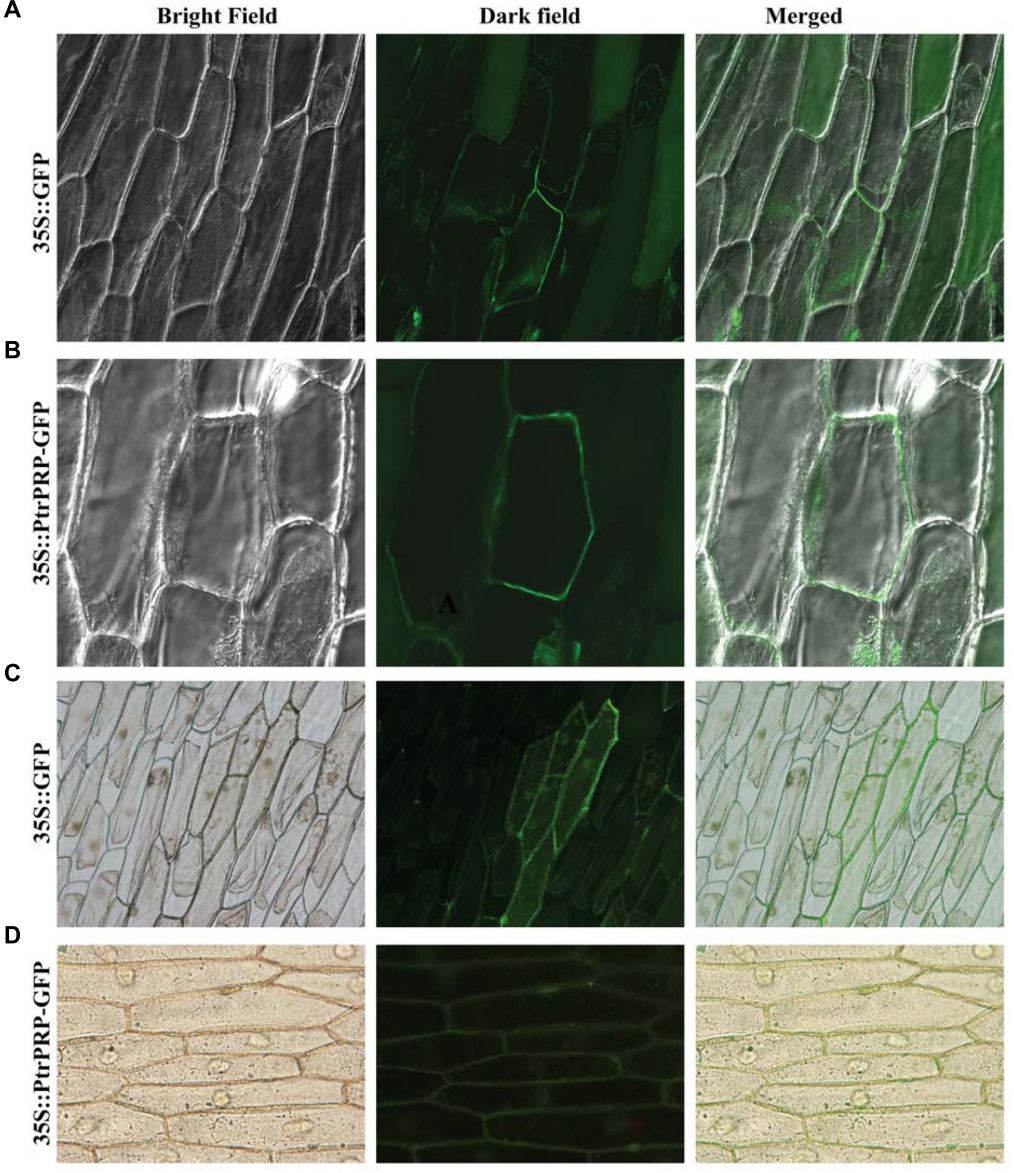
**Subcellular localization analysis of PtrPRP.** GFP **(A,C)** or PtrPRP-GFP **(B,D)** was transiently expressed in onion epidermal cells by *Agrobacterium*-mediated transfection, and the onion cells were observed under a confocal microscope **(A,B)** or fluorescence microscope **(C,D)**. Images taken under bright (left) or dark (middle) field were shown, while the merged images are shown on the right.

Subcellular localization of PtrPRP was investigated by constructing a fusion protein of PtrPRP without the stop codon and GFP, driven by *CaMV 35S* promoter, using the GFP vector as a control. Microscopic observation showed that green fluorescence could be observed in the whole cells when the control plasmid was transiently expressed (**Figures 3A,C**). However, when the fusion protein was expressed in the onion epidermis, green fluorescence was predominantly observed on the outer surface of the cells (**Figures 3B,D**), which may include the plasma membrane (PM), internal membranes and PM/cell wall interphase. However, the exact localization to a certain position of *PtrPRP* remains to be determined.

### Expression Profiles of PtrPRP in Different Tissues and Under Various Treatments

**FIGURE 4:**
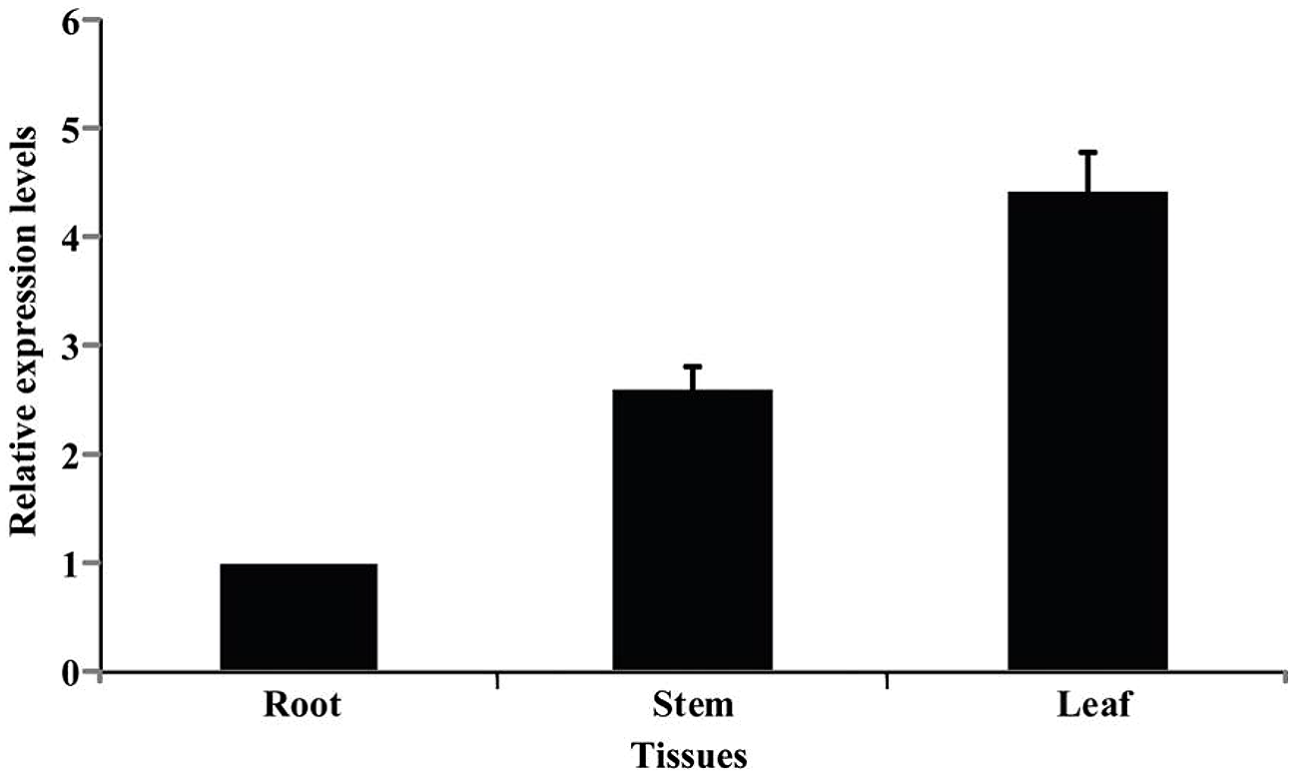
**Expression analysis of PtrPRP in three different tissues, including root, stem, and leaf, as revealed by qPCR.** Expression level of *PtrPRP* in the root was set as 1, and those in other tissues were calculated accordingly.

**FIGURE 5:**
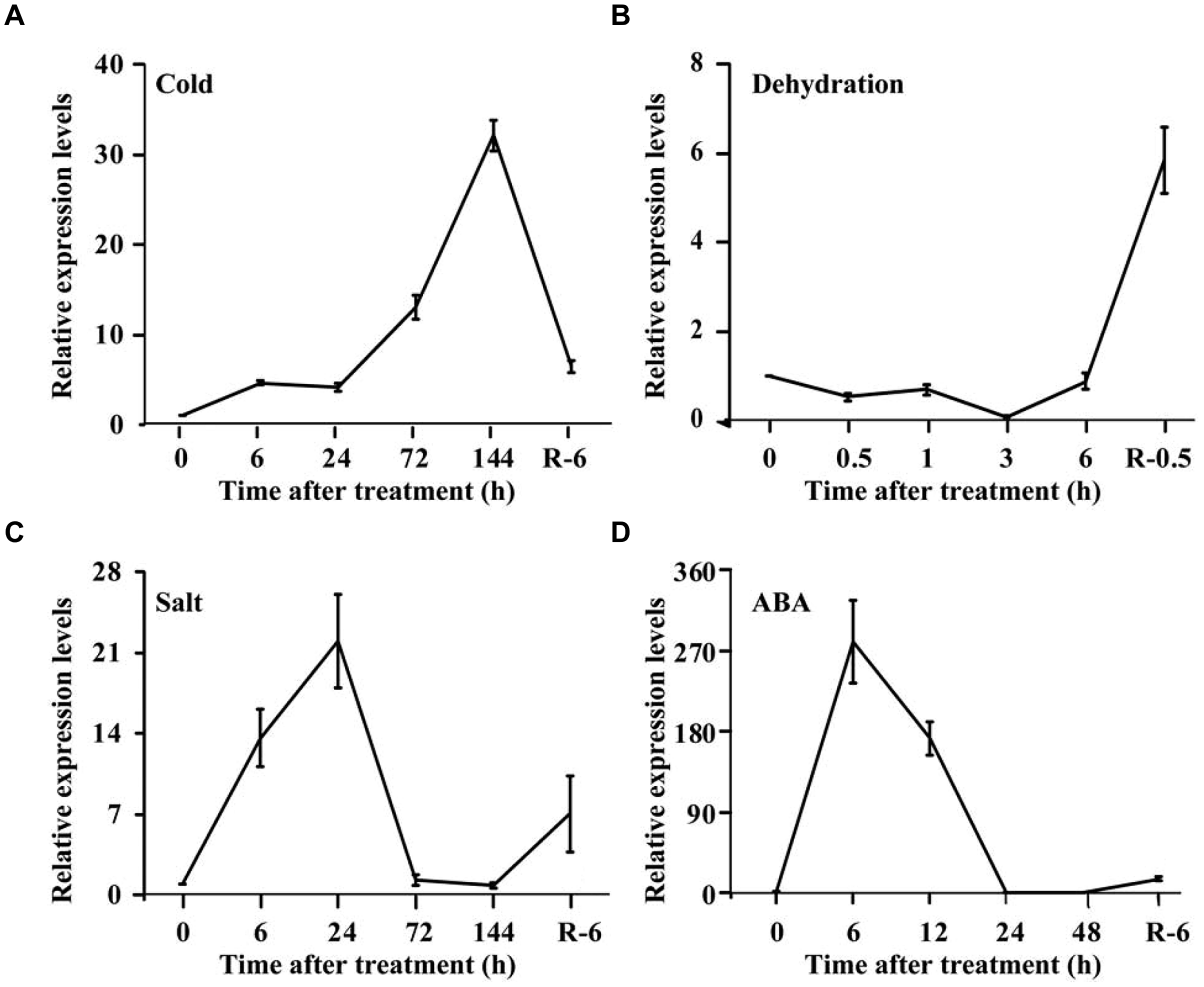
**Expression analysis of PtrPRP in response to various treatment, including cold **(A)**, dehydration **(B)**, salt **(C)**, and ABA **(D)**.** Expression levels of PtrPRP at the onset of treatment (0 h) were set as 1, and those at other time points were calculated accordingly. R indicates recovery after the corresponding treatments.

Spatial expression of *PtrPRP* in three tissues, leaf, root, and stem, was assessed by qPCR. *PtrPRP* was constitutively expressed in the three tissues, but their transcript levels varied among each other (**Figure 4**). The highest mRNA abundance was detected in the leaf, whereas root showed the lowest expression level among the three tissues. In addition, expression patterns of *PtrPRP* in response to cold, dehydration, salt, and ABA treatments were examined. Under exposure to cold, transcript level of *PtrPRP* was quickly induced within 6 h, and then progressively accumulated to reach the highest level at 144 h, when the expression level was elevated by more than 30-folds compared with that at 0 h. However, when the cold treatment was removed, transcript level of *PtrPPR* was sharply decreased (**Figure 5A**). Dehydration treatment led to gradual reduction of *PtrPRP* mRNA levels up to the lowest value at 3 h, followed by an accretion to the basal level at 6 h. Surprisingly, rehydration for 6 h resulted in a notable induction of *PtrPRP* (**Figure 5B**). Steady-state mRNA level of *PtrPRP* was up-regulated by nearly 14-folds within 6 h of salt treatment, and continued to rise until reaching the peak value at 24 h, followed by a sharp decrease to basal level at 72 and 144 h. Relief of the salt stress for 6 h led to a sevenfolds elevation of the transcript level (**Figure 5C**). Expression pattern of *PtrPRP* in response to exogenous ABA application was similar to that under salt treatment, except the greater induction within the first two time points. Removal of ABA also led to an up-regulation of *PtrPRP* transcript (**Figure 5D**).

### Production of Trifoliate Orange RNAi Lines with Knock-down of PtrPRP

**FIGURE 6:**
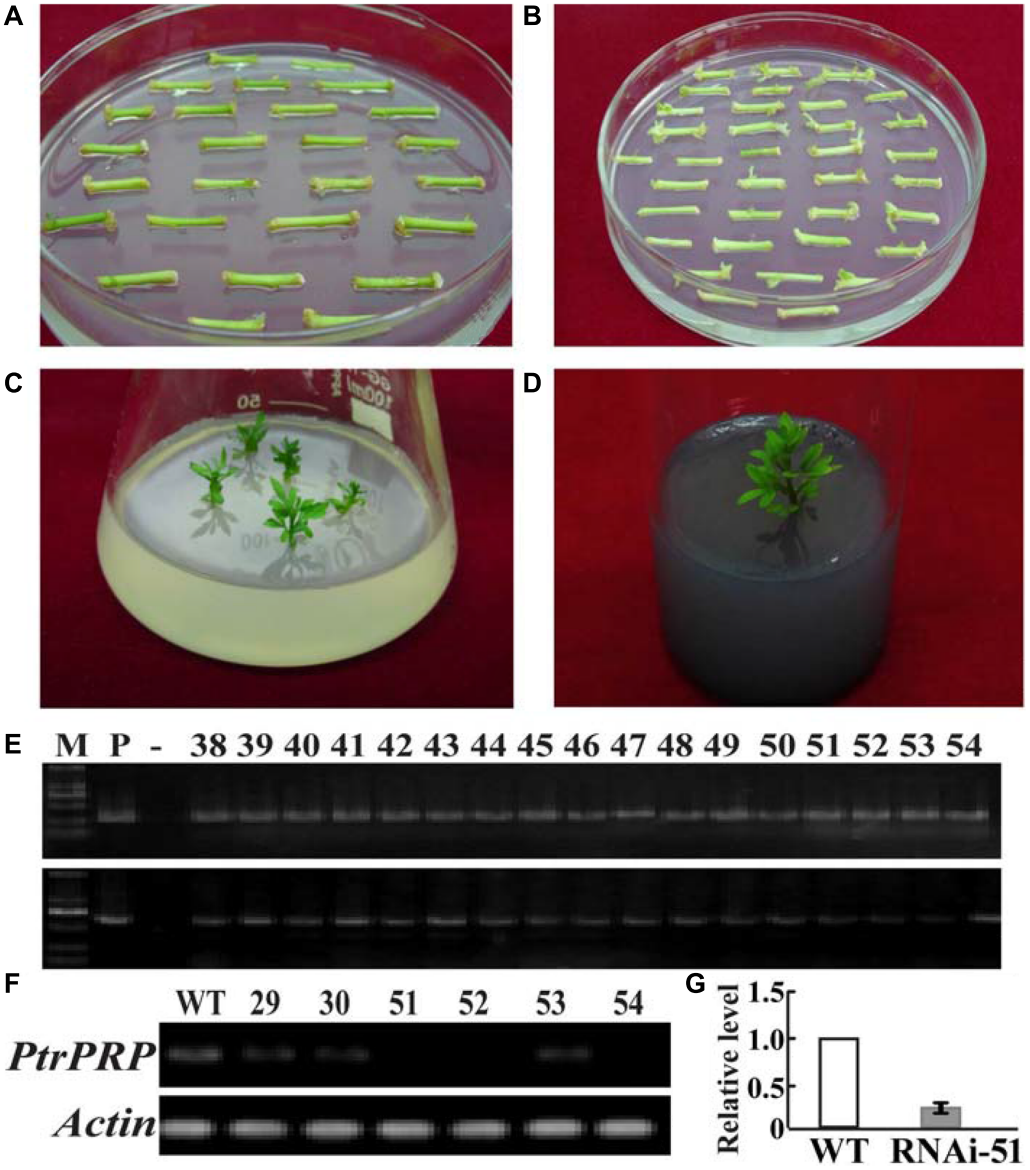
**Transformation, regeneration, and characterization of trifoliate orange transgenic plants. (A,B)** Culture of the stem segments on selection medium for 30 d **(A)** and 60 d **(B)**, respectively. **(C)** Regeneration of kanamycin -resistant shoots on the selection medium. **(D)** A rooting plant on the root-inducing medium. **(E)** Genomic PCR of the kanamycin-resistant plants using designed primers specific to PtrPRP (upper) and NPTII (bottom), respectively. **(F)** Expression analysis of *PtrPRP* in six positive transgenic plants, as revealed by RT-PCR. Actin gene was used as an internal control. **(G)** Analysis of *PtrPRP* expression level in RNAi-51 using qRT-PCR.

To elucidate the role of *PtrPRP* in cold tolerance, RNAi strategy was used to suppress *PtrPRP* in trifoliate orange. For this purpose, a 253-bp cDNA fragment displaying lower degree of sequence conservation among C-terminal ends of the PRPs was used to construct *PtrPRP*-RNAi vector, which was then transferred into trifoliate orange via *Agrobacterium*-mediated transformation (**Figures 6A–D**). Kanamycin-resistant plants were confirmed to be positive using genomic PCR (**Figure 6E**). Semi-quantitative RT-PCR assay indicated that among the positive lines *PtrPRP* was successfully down-regulated in three lines, and the greatest suppression was observed in lines #51 and #52, which were hereafter designated as RNAi-51 and RNAi-52, respectively (**Figure 6F**). Expression of *PtrPRP* in RNAi-51 was also checked using qRT-PCR, and the results confirmed that PtrPRP is trully knocked down, purporting the semi-quantitative RT-PCR data (**Figure 6G**). The two RNAi lines, RNAi-51 and RNAi-52, showed no difference in plant morphology in comparison with the WT.

### Knock-down of PtrPRP Confers Sensitivity to Chilling Stress

**FIGURE 7:**
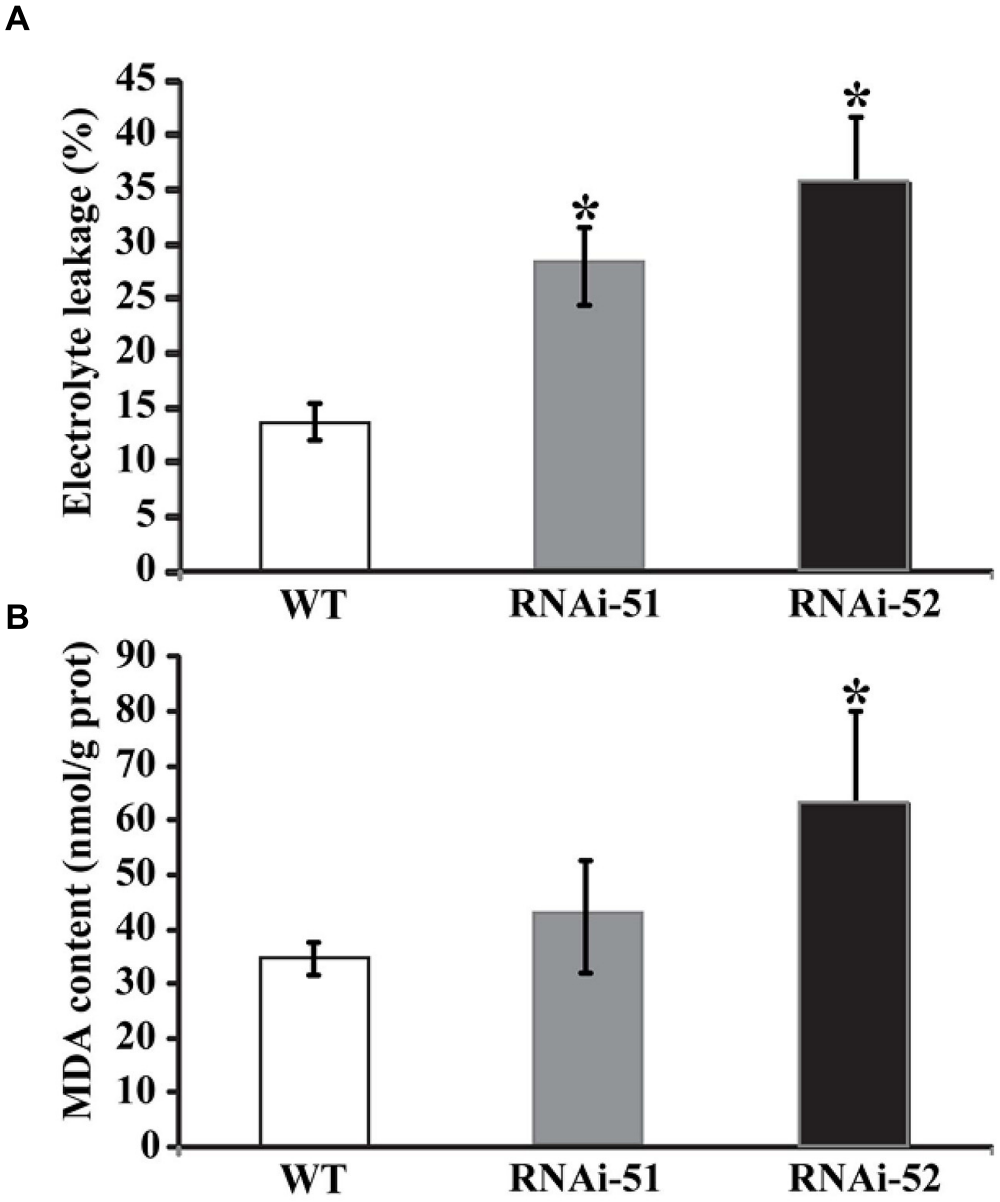
**Analysis of electrolyte leakage (EL) **(A)** and malondialdehyde (MDA) **(B)** in wild type (WT) and two RNAi lines after cold treatment.** Asterisks indicate that the values of corresponding transgenic lines are statistically significantly different from that of WT (*P < 0.05).

The two RNAi lines and WT were subjected to a chilling temperature at 0°C for 48 h so as to investigate the impact of silencing *PtrPRP* on cold tolerance. We examined EL and MDA, two critical parameters that have been widely used for evaluating the stress tolerance in earlier studies (Peng et al., 2012, 2014). After the chilling treatment, EL and MDA of both RNAi lines were significantly higher than those of WT, indicating that more severe damages have been imposed on the RNAi lines compared to the WT (**Figures 7A,B**).

### The RNAi Lines Accumulate More ROS but Less Proline

**FIGURE 8:**
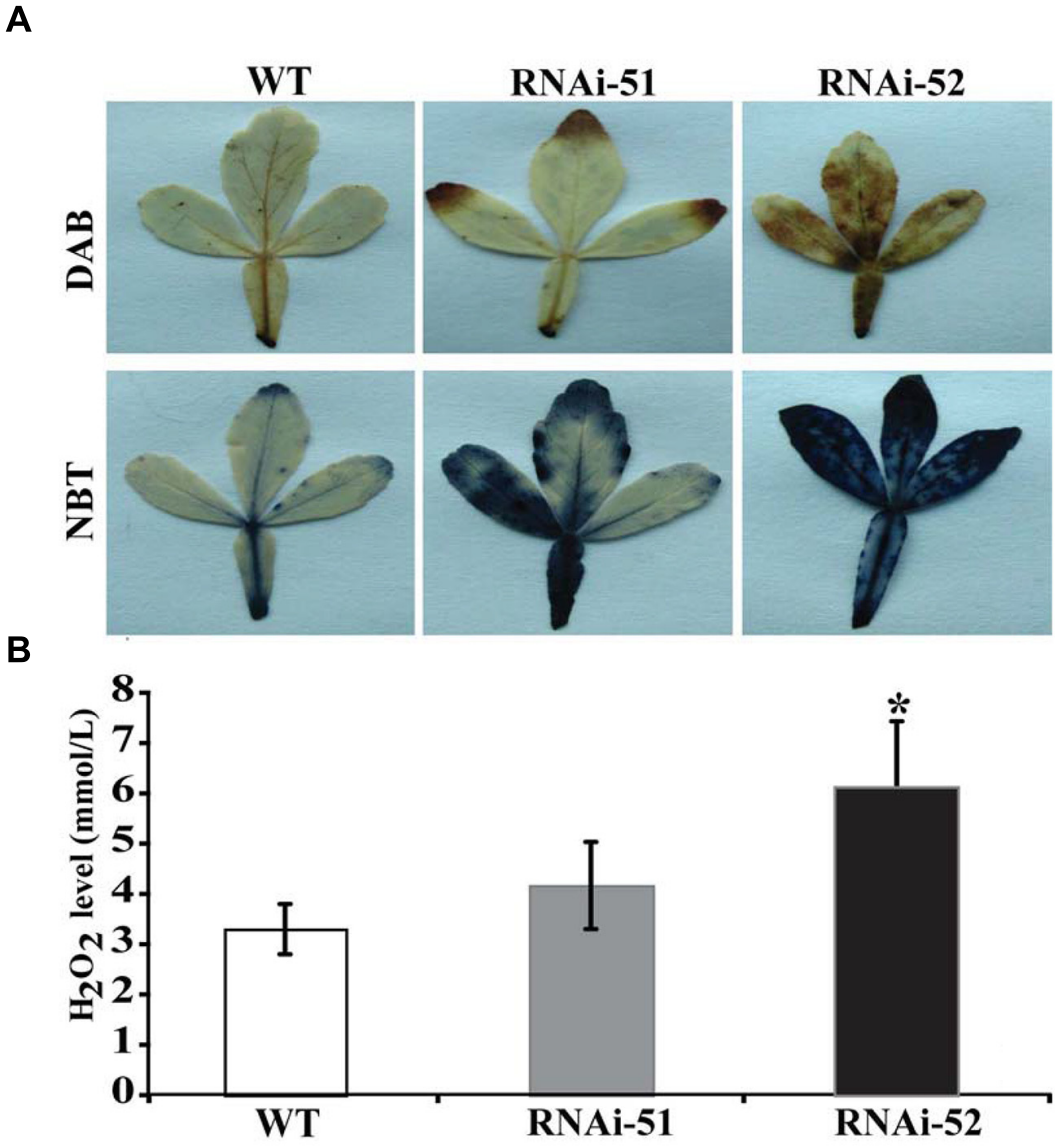
**Histochemical staining and quantitative measurement of ROS. (A)** Histochemical staining with DAB (upper) and NBT (lower) in WT and two RNAi lines after cold treatment. **(B)** Quantitative measurement of H_2_O_2_ in WT and the RNAi lines. Asterisks indicate that the values of corresponding transgenic lines are statistically significantly different from that of WT (*P < 0.05).

**FIGURE 9:**
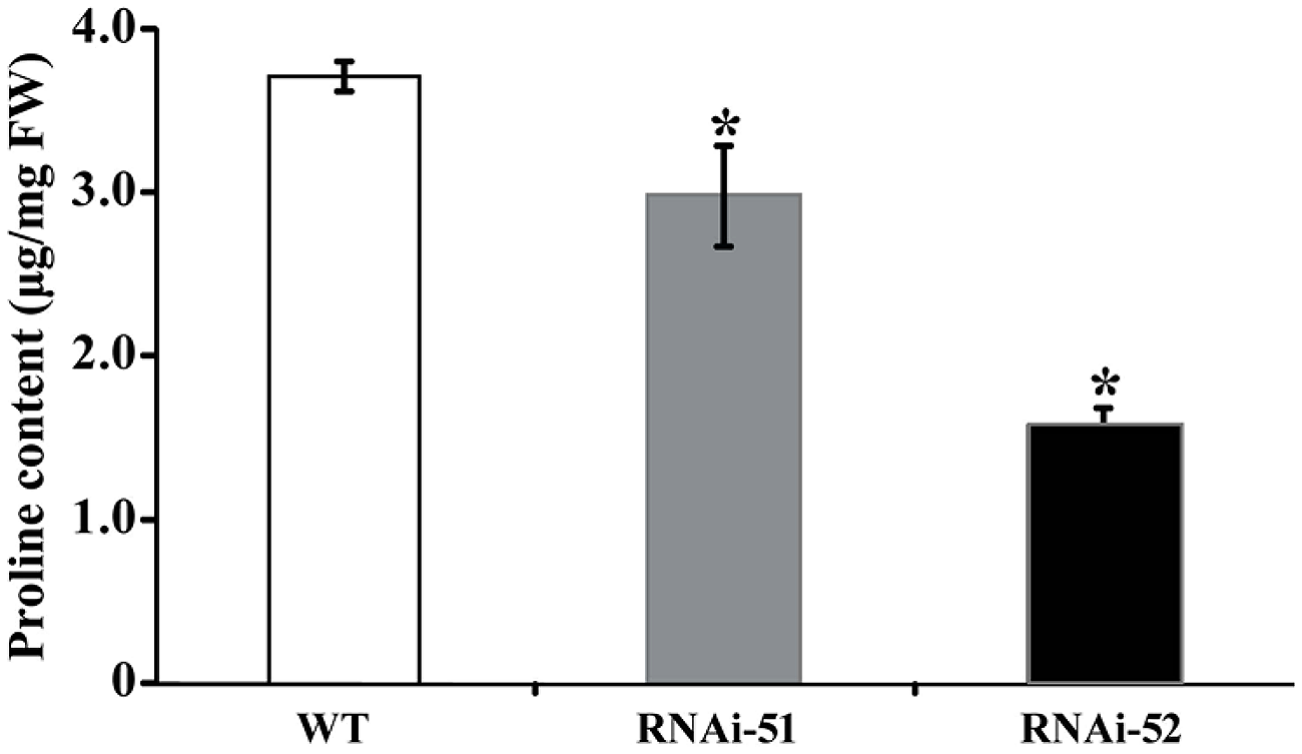
**Analysis of proline levels in WT and two RNAi lines after cold treatment.** Asterisks indicate that the values of corresponding transgenic lines are statistically significantly different from that of WT (*P < 0.05).

Electrolyte leakage and MDL are indirect indices for oxidative stress that is primarily caused by excessive accumulation of ROS. The drastic difference in EL and MDA levels between RNAi lines and WT prompted us to check ROS status of the tested lines after cold treatment. We first used histochemical staining with DAB and NBT to reveal *in situ* accumulation of H_2_O_2_ and O_2_^-^, respectively, in the cold treated leaves. This method is valid as the ROS levels can be directly disclosed based on the color of reaction. As shown in **Figure 8A**, conspicuous difference in the staining patterns was observed between WT and the RNAi lines. The leaves of RNAi lines were stained by both DAB and NBT in deeper manner or the areas of staining were larger, indicating that the RNAi lines produced more ROS after cold treatment compared with the WT. The staining was partly confirmed by quantitative measurement of H_2_O_2_ using a specific kit designed for it (**Figure 8B**). We also measured proline contents in the RNAi lines and WT after cold treatment, as this compound has been considered as an important metabolite indicating the relevance to stress tolerance. As shown in **Figure 9**, the WT accumulated more proline in comparison with the two RNAi lines.

## Discussion

Although HyPRPs have been reported in many plants, the precise functions of HyPRPs were still poorly elucidated, especially in woody plants. Here we report molecular cloning and functional characterization of *PtrPRP*, a *HyPRP* gene from *Poncirus trifoliata*. Since genome of *P. trifoliata* has not been sequenced, it remains to be determined whether *P. trifoliata* has more PRP members at this stage. By search against the whole genome sequences of *Citrus sinensis*, a closely related species of *P. trifoliata* we found four putative PRPs (CsPRPs), among which CsPRP1 shares the highest similarity with PtrPRP. It suggests that *P. trifoliata* may possibly contain other PRPs, but this assumption needs to be verified in the future. In PtPRP, proline residues account for 21% of the total amino acids of PtrPRP, allowing us to believe that PtrPRP is a PRP. Of note, the prominent abundance of proline residues at the N terminus may be possibly associated with the targeting of this protein, as the presence of hydrophobic proline-rich (HPR) motif has been suggested to be necessary and sufficient for the intracellular targeting of a temperature-induced lipocalin in *Arabidopsis* (AtTIL; Hernaìndez-Gras and Boronat, 2015). So far, PRPs have been previously categorized into five groups based on signature motifs (Gothandam et al., 2010), but PtrPRP belongs to none of them. However, sequence alignments between PtrPRP and HyPRPs from several plants revealed that PtrPRP has the conserved 8 CM domain, which is a typical signature of HyPRPs, but not present in PRPs (Josè-Estanyol et al., 2004), which indicates that PtrPRP is possibly considered as a HyPRP. In addition, since PtrPRP contains the 8 CM domain it should be categorized into the Class B HyPRPs.

Expression of *PRPs* was shown to be associated with development of various tissues and exhibited temporal and spatial expression patterns (Vignols et al., 1999; Gothandam et al., 2010; Neto et al., 2013). Transcripts of *ZmHyPRP* from maize were specifically observed in the immature embryos (Josè-Estanyol et al., 1992), while a *HyPRP* gene of strawberry was exclusively found in mature fruits (Blanco-Portales et al., 2004). Hong et al. (1989) reported that soybean contained three PRPs, *SbPRP1*, *SbPRP2*, and *SbPRP3*. *SbPRP1* was highly expressed in mature hypocotyl, root, and immature seed coat. *SbPRP2* was the predominant form of transcripts in the apical hypocotyl and young suspension culture cells, while the transcripts of *SbPRP3* accumulated mainly in aerial parts, especially in leaves. In a recent study, Neto et al. (2013) expanded the soybean HyPRP proteins to 35 members, which exhibited variable expression patterns in six vegetative organs, root, and root tip, nodule, leaves, green pods, flower, and apical meristem. Some of them were not detected in any tissue, while others were expressed in specific organs. Interestingly, four soybean *HyPRPs* were almost exclusively highly expressed in leaves, consistent with our finding on *PtrPRP*, which displayed the highest expression level in leaf. These findings suggest that plant *PRPs* may exhibit spatial expression patterns, which implies that the PRPs may function in different ways among various tissues.

In this study, we also noticed that PtrPRP was remarkably induced by cold and salt, but underwent minor change under dehydration. Of note, when the abiotic stresses were removed, *PtrPRP* showed opposite expression patterns in comparison with those under the corresponding stresses. These results indicate that *PtrPRP* was sensitively responsive to the stresses, but the response is different among various environmental stimuli. This phenomenon has been also observed in other PRP gene. For example, *OsPRP1* exhibited different or even reverse expression patterns under various stress factors (Wang et al., 2006). In another work, *CcHyPRP* transcripts of pigeonpea (*Cajanus cajan* L.) were shown to be enhanced in response to treatments with PEG, NaCl, heat (42°C), and cold (Priyanka et al., 2010). These findings suggest that plant PRP genes are differentially regulated under abiotic stresses and that different member of the PRP family may possibly play specific roles in mediating abiotic stress tolerance. In addition, *PtrPRP* was quickly and sharply induced by ABA, implying that *PtrPRP* is an ABA-responsive gene. However, expression of PtrPRP under ABA treatment was consistent with *RePRP* of rice and *CcHyPRP* of pigeonpea, but it was contradictory to *OsPRP*, which was repressed by ABA (Akiyama and Pillai, 2003; Priyanka et al., 2010; Tseng et al., 2013). However, whether *PtrPRP* functions in an ABA-dependent manner needs to be elucidated.

Down-regulation of gene expression via RNAi at post-transcriptional level has been widely used as an alternative approach for functionally characterizing genes involved in biotic and abiotic stress tolerance, because RNAi plants may clearly display altered phenotype or metabolic disorders (Mao et al., 2007; Tardieu and Tuberosa, 2010; Duan et al., 2012; Huang et al., 2013; Saurabh et al., 2014). In keeping with this, herein we employed RNAi approach to knock down *PtrPRP* in trifoliate orange so as to elucidate the function of this gene in cold stress tolerance. After 48 h of cold treatment, EL and MDA content, two parameters for membrane integrity, in the RNAi lines were higher than in the control, suggesting that knock-down of *PtrPRP* led to severer membrane damage. It was reasonable because PtrPRP was localized in the membrane. In higher plant, cell membrane encloses cytoplasm and various organelles; thus maintenance of cell membrane integrity is critical for plant to overcome the physiological and biochemical changes induced by cold stress. The well-documented injuries caused by cold are largely due to osmotic and oxidative stresses that pose serious threat to membrane integrity (Welti et al., 2002; Peng et al., 2014). Therefore, when challenged by low temperature stress, biosynthesis and degradation of some related proteins might be expedited to stabilize the integrity of cellular membranes against cold injury (Achard et al., 2008). PRPs are proposed to play an integral role in consolidation of extracellular matrix structure of plant cells, which is an important approach for elevating mechanical strength to the cell wall. In this case, the PRPs may confer the integrity of plant membranes and promote the structure maintenance of organs (Gothandam et al., 2010). Therefore, it is reasonable to assume that when this protein accumulation was suppressed by RNAi, the protective roles of PtrPRP in membrane integrity might be impaired, which in turn leads to greater membrane damage, as revealed by higher EL and MDA levels.

Proline has been documented to act as an important compounds involved in stress tolerance as they can serve as an osmolyte to optimize the physical structure of cell membrane for proper cellular function (Duncan and Widholm, 1987; Akiyama and Pillai, 2003; Rodriguez and Redman, 2005; Battaglia et al., 2007). The proline level has been shown to be associated with the magnitude of a plant to alleviate membrane damages and enhance cell viability via mitigation of osmotic stress and reduction of MDA production (Ozden et al., 2009), which agrees with our data that MDA content in the two RNAi lines was higher than in the WT. These illustrations suggest that accumulation of less proline may lead to disordered osmotic adjustment, which constitutes a mechanism underlying the cold sensitivity of the RNAi lines. Except acting as an osmolyte, PtrPRP may also contribute to scavenging of ROS, which are by-products of various metabolic processes (Mittler, 2002; Finkel, 2011). Excessive accumulation of ROS is known to cause oxidative damages to cells and thus impairs normal physiological or biological processes. Plant has developed either enzymatic or non-enzymatic antioxidant defense systems to maintain ROS homeostasis and to alleviate the burst of ROS. The non-enzymatic system is composed of a number of compounds that are generally known as low MW antioxidants, such as betaine and proline (Rodriguez and Redman, 2005; Fu et al., 2011; Hayat et al., 2012; Keunen et al., 2013; Peng et al., 2014). In earlier studies, exogenous application of proline has been shown to mitigate ROS production and confer enhanced stress tolerance, implying that proline plays a significant role in ROS scavenging (Hoque et al., 2007; Kaul et al., 2008). Hong et al. (2000) suggested that the role of proline as a ROS scavenger is more important than its role as an osmolyte under stress conditions, further supporting the crucial value of proline in detoxifying ROS. In this work, we found that suppression of *PtrPRP* in the RNAi lines was accompanied by a noticeable reduction of proline, concurrent with the prominent elevation of ROS levels. These findings indicate that *PtrPRP* may function in cold tolerance by influencing ROS homeostasis due to, at least in part, the role of proline as an efficient antioxidant for ROS detoxification. Nevertheless, why and how knock-down of PtrPRP caused a decrease of proline in the RNAi lines remained elusive and needs to be investigated in the future.

## Conclusion

Our data demonstrated that PtrPRP of *P. trifoliata* is a hybrid-PRP. *PtrPRP* expresses in various tissues and responds to abiotic stimuli, such as cold, salt, and ABA. RNAi-based knock-down of *PtrPRP* conferred enhanced cold sensitivity of the RNAi lines at chilling temperature, as manifested by severer membrane damage, accumulation of more ROS and less production of proline under cold stress. Therefore, PtrPRP may contribute to cold tolerance via modulation of proline, an important osmolyte and ROS scavenger.

## Conflict of Interest Statement

The authors declare that the research was conducted in the absence of any commercial or financial relationships that could be construed as a potential conflict of interest.

## Footnotes

1 http://us.expasy.org

2 http://phylogeny.lirmm.fr/phylo_cgi/index.cgi

3 http://www.jalview.org/

4 http://www.cbs.dtu.dk/services/SignalP/

5 http://hc.ims.u-tokyo.ac.jp/iPSORT/
